# Incidence of fracture of ProDesign Logic system instruments: A cross-sectional retrospective study

**DOI:** 10.1038/s41598-022-11605-x

**Published:** 2022-05-12

**Authors:** Ricardo Machado, Claudemir de Souza Júnior, Bruna Larissa Mendes, Viviane Vieira, Stella Maria Glaci Reinke, Ana Cristina Gonçalves Kovalik, Eduardo Donato Eing Elgelke Back, Daniel Comparin

**Affiliations:** 1 Department of Endodontics, School of Dentistry, Regional University of Blumenau – FURB, Blumenau, Santa Catarina Brazil; 2 Private Clinical Practice, Foz do Iguaçú, Paraná Brazil; 3 School of Dentistry, Regional University of Blumenau – FURB, Blumenau, Santa Catarina Brazil; 4 Department of Periodontology, School of Dentistry, Regional University of Blumenau – FURB, Blumenau, Santa Catarina Brazil; 5 Private Clinical Practice, Joinville, Santa Catarina Brazil; 6 Department of Endodontics, School of Dentistry, Paranaense University – UNIPAR, Francisco Beltrão, Paraná Brazil; 7 Present Address: Rua Brasília, n. 300, Apto.503, Centro, Navegantes, Santa Catarina 88370-100 Brazil

**Keywords:** Dental diseases, Dental caries, Pulpitis, Diseases, Infectious diseases, Bacterial infection

## Abstract

The aim of the present study was to analyze the incidence of fracture of ProDesign Logic system instruments in endodontic treatments performed by a specialist, in addition to identifying the dental group, arch, and the root canal thirds in which the fractures occurred more frequently. Digital radiographs and medical records were initially analyzed and resulted in the selection of 561 teeth (1302 canals) treated between 2018 and 2020, using the ProDesign Logic system instruments. These data were reassessed to determine the occurrence of fractures and identify the dental group and root canal thirds in which they occurred. Then, the data were statistically analyzed using the Fisher’s Exact Test (p < 0.05). The general fracture rates were 8.5 and 3.69%, considering the number of teeth and canals treated, respectively. Mandibular first molars were the teeth most associated with the occurrence of fractures (19.1%). When the arches were compared, there was no statistical difference regarding the number of fractures in the different root canal thirds (p = 0.307). However, they were more frequent in the apical third in both arches (p = 0.000). The incidence of fracture of ProDesign Logic system instruments was relatively high and occurred more frequently in the apical third of molars.

## Introduction

The essential goal of endodontic treatment is to maintain or restore the health of periapical tissues. In vital teeth, the pulp is removed, and the root canals are cleaned, shaped and hermetically filled with a biocompatible material. Whereas the periapical tissues are not compromised, the procedure is performed with the aim of maintaining their integrity. On the other hand, pulp necrosis completely compromises its cells, and allows infection of the root canal system (RCS) to occur. In these cases, the purpose of endodontic treatment is to prevent the development of a chronic periapical inflammation or restore the health of the local tissues^1^.

Complete eradication of the endodontic infection is unfeasible, mainly due to bacterial resistance^2^, anatomical complexity^3,4^ and the presence of extraradicular biofilms^5^. However, high success rates in Endodontics are primarily based on the control of intracanal infection^6^.

Biomechanical preparation is the main procedure responsible for reducing the microbial contingent to levels that favor the maintenance of periapical health or its restoration to normality. The procedure is performed by means of instruments and auxiliary chemical substances responsible for the mechanical and chemical cleaning of the RCS, respectively^7,8^. Irrigation also plays a role in the physical cleaning action based on the flow and reflux of the irrigant solution^9^.

The technical-scientific evolution experienced in the field of Endodontics in the last few decades has allowed that professionals to have a glimpse of a new horizon on procedures for mechanical cleaning of the root canal. New kinematics and instruments have made it possible to perform faster and more comfortable endodontic treatments and retreatments for both patients and professionals^10–12^, in addition to minimizing the possibility of errors and accidents^10,13,14^ and facilitating the obturation procedures^15^. However, fractures that mainly occur due to cyclic fatigue and/or torsion, are one of the main concerns associated with the use of this type of instrument^16–18^.

In curved canals, NiTi files used in continuous rotation are subjected to two types of antagonistic stresses. The portions of the instrument that act on the outside and inside of the curvature undergo tension and compression, respectively. At each rotation, there is a complete cycle of tension and compression that can cause instrument fracture by cyclic fatigue. Torsional fractures occur when the tip of the file is immobilized, while the force applied to displace it continues (torque). When the plastic limit of the instrument is overcome by this force, fracture occurs. Factors such as design, NiTi alloy used for manufacturing, kinematics and number of uses of the file, also have a decisive influence on the occurrence of this “undesirable event”^16–18^.

The ProDesign Logic system (Easy Equipamentos Odontológicos, Belo Horizonte, Minas Gerais, Brazil) was developed for the purpose of making more conservative biomechanical preparations by means of less wear on the peri-cervical dentin. The instruments are machined from a conventional NiTi alloy, later submitted to heat treatment (CM, *controlled memory*), which gives them greater flexibility and resistance to cyclic and torsional fatigue^19,20^. The system consists of instruments specially designed for performing the glide path—#25, #30, #35, #40, #45 and #50 (0.01 taper) and #15 (0.03 taper); preparation instruments (shape/finishing)—#25, #30, #35 and #40 (0.03 and 0.05 tapers), and additional instruments—#15 (0.05 taper) and #25 (0.04 and 0.06 tapers). All are manufactured in lengths of 21 and 25 mm, and feature a quadruple (0.01 taper), double (0.03, 0.05 and 0.06 taper), triple (#25/0.04) and quadrangular helix-shaped cross section (#15/0.03 and #15/0.05)^21^.

Menezes et al.^19^ have demonstrated that ProDesign Logic system files (Easy Equipamentos Odontológicos) had greater resistance to cyclic fatigue and performed faster preparations after the glide path, when compared with the instruments of the WaveOne Gold system (Dentsply-Maillefer, Ballaigues, Switzerland). Other favorable properties, such as maintaining the original shape of the root canal, good cutting power and resistance to cyclic fatigue, in addition to great flexibility, have also been demonstrated, and these features are mainly attributed to the heat treatment to which these instruments have been subjected^22–25^. However, up to now, no research has been conducted to analyze the incidence of fracture of ProDesign Logic system files (Easy Equipamentos Odontológicos) in clinical use.

The aim of the present study was to investigate the incidence of fracture of ProDesign Logic system instruments (Easy Equipamentos Odontológicos) in endodontic treatments performed by a specialist, in addition to identifying the dental group, arch, and root canal thirds in which the fractures occurred more frequently.

## Materials and methods

### Ethical approval and informed consent

This study was authorized and approved by the Research Ethics Committee of the Paranaense University—UNIPAR, Francisco Beltrão, Paraná, Brazil (n. 34388520.7.0000.5370), and it was performed considering the principles of the World Medical Association Declaration of Helsinki “Ethical Principles for Medical Research Involving Human Subjects”, (amended in October 2013). Informed consent was obtained from each patient or guardians of patients under 18 years of age, who participated in the study.

### Clinical protocol

Once the need for endodontic treatment was confirmed by means of anamnesis and clinical-radiographic examination, anesthesia was administered, rubber dam was placed and endodontic opening was performed with a spherical drill No. 1012, 1014 or 1016 (KG Sorensen, Barueri, São Paulo, Brazil), depending on the coronal volume. After the initial exploration of the root canal with a K-Flexofile #10 or #15 (Dentsply-Maillefer), preparation of the entrance and glide path were performed with a Largo No. I, II or III and with a ProDesign Logic instrument #15/0.01 or #25/0.01 (Easy Equipamentos Odontológicos), respectively, considering clinical-radiographic characteristics of the root canals. Then, the cervical and middle thirds were prepared with a ProDesign Logic instrument #25/0.06 or #25/0.05 (Easy Equipamentos Odontológicos), also based on their anatomy. The working length was established at -0.5 mm of the apical foramen, by using an electronic apex locator (Root ZX, J. Morita, Japan), followed by determining the anatomical diameter, by using first series K-Flexofiles (Dentsply-Maillefer) in ascending order. Once the anatomical diameter was determined, the instrumentation amplitude was established in cases of vitality and pulp necrosis (Table 1), and conducted using 2.5 ml of 2.5 and 5.25% sodium hypochlorite (Fórmula & Ação, São Paulo, São Paulo, Brazil) respectively, after every use or exchange of files. In case there were fractures, periapical radiographs were taken to confirm their presence, location and they were recorded (tooth/arch, canal and third). Each instrument was used in a maximum of 4 molars, 10 premolars with 2 canals, 20 and 25 premolars and anterior teeth with a single canal, respectively. In severely curved and/or atresic canals, the files were used only once.

**Table 1:** Planning the amplitude of the chemomechanical preparation based on the anatomical diameter in vital and necrotic teeth.

Anatomical diameter*	Final file used during chemomechanical preparation (vital teeth)**	Final file used during chemomechanical preparation (necrotic teeth)**
10	25/0.01	25/0.04
15	25/0.04	30/0.01
20	30/0.01	35/0.01
25	35/0.01	40/0.01
30	40/0.01	45/0.01
35	45/0.01	50/0.01

*K-Flexofiles.

**NiTi file of the ProDesign Logic system (Easy Equipamentos Odontológicos).

### Data collection

Digital radiographs and medical records were initially analyzed and resulted in the identification of 561 teeth (1302 canals) treated by a specialist between 2018 and 2020, according to the clinical protocol described above. Then, this material was evaluated again for the purpose of tabulating the following data: tooth, quantification and identification of the canals, in addition to the incidence of fracture of instruments, or not. If positive, the canal and the root canal third in which the fracture had occurred were recorded.

### Statistical analysis

The data obtained were analyzed using SPSS 25 software (IBM Corp., Armonk, NY, United States [https://www.ibm.com/support/pages/downloading-ibm-spss-statistics-25]) using Fischer’s exact test with a significance level of 5% (p < 0.05). The complete data can be accessed by the link https://1drv.ms/x/s!AnuvHAhxDtNGgsAWd5p3-roKl9gs4Q?e=0NzcBg.

## Results

The number of teeth and canals treated, as well as the number of fractures per arch are shown in Table 2.

**Table 2:** Number of teeth, canals, and fractures per arch.

Dental group	Quantity of canals	Maxillary arch			Mandibular arch		
		Number of teeth	Number of canals	Number of fractures	Number of teeth	Number of canals	Number of fractures
Central incisors	1	34	34	0	8	8	0
	2	0	0	0	1	2	0
Lateral incisors	1	36	36	0	5	5	0
Canines	1	11	11	0	9	9	0
First premolars	1	6	6	0	13	13	0
	2	51	102	3	1	2	0
	3	1	3	0	0	0	0
Second premolars	1	22	22	0	25	25	0
	2	43	86	1	2	4	0
First molars	1	0	0	0	0	0	0
	2	0	0	0	0	0	0
	3	56	168	4	49	147	7
	4	28	112	4	40	160	10
Second molars	1	2	2	0	1	1	0
	2	5	10	0	11	22	0
	3	38	114	7	40	120	6
	4	6	24	1	5	20	3
Third molars	1	0	0	0	0	0	0
	2	0	0	0	3	6	0
	3	3	9	1	5	15	1
	4	0	0	0	1	4	0
Total	–	342	739	21	219	563	27

Table 3 shows the number and percentage of fractures according to the number of teeth and canals treated per arch.

**Table 3:** Number and percentage of fractures according to the number of teeth and canals treated per arch.

	Maxillary arch	Mandibular arch	Total	Percentage of fractures
Number of fractures/teeth treated	21/342 (6.14%)	27/219 (12.32%)	48/561	8.50
Number of fractures/ canals treated	21/739 (2.84%)	27/563 (4.79%)	48/1302	3.69

Disregarding the third molars due to the low number of treated teeth, it was observed that the fractures occurred more frequently in the mandibular first molars (19.10%) and maxillary (15.70%) and mandibular (15.80%) second molars (Table 4).

**Table 4:** Number and percentage of teeth in which fractures occurred.

Tooth	Arches	Teeth treated	Incidence of fractures	Percentage of fractures considering total number of teeth treated	Percentage of fractures in relation to dental group itself	Percentage in relation to general number of fractures
First premolars	Maxillary	58	3	0.500	5.2	6.20
	Mandibular	14	0	0	0	0
Second premolars	Maxillary	65	1	0.20	1.50	2.10
	Mandibular	27	0	0	0	0
First molars	Maxillary	84	8	1.40	9.50	16.70
	Mandibular	89	17	3	19.10	35.40
Second molars	Maxillary	51	8	1.40	15.70	16.70
	Mandibular	57	9	1.60	15.80	18.70
Third molars	Maxillary	3	1	0.20	33.40	2.10
	Mandibular	9	1	0.20	11.10	2.10
–	Total	–	48	8.50	–	100

When comparing the arches, there was no statistically significant difference (p = 0.307) with reference to the occurrence of fractures in both root canal thirds (middle: p = 0.434/apical: p = 0.633). However, a significantly higher number of fractures were observed in the apical third (p = 0.000), in both the mandibular (p = 0.006) and maxillary arches (p = 0.002). There were no fractures in the cervical third (Table 5).

**Table 5:** Distribution and percentage of fractures considering arch and root canal third.

Arch	Root canal third			Total (n./%)	p-value*
	Cervical (n./%)	Middle (n./%)	Apical (n./%)		
Mandibular	0/0%	8/29.60%	19/70.40%	27/100%	0.006
Maxillary	0/0%	5/23.80%	16/76.20%	21/100%	0.002
Total	0/0%	13/27.10%	35/72.90%	48/100%	0.000
p-value*	–	0.434	0.633	0.307	–

*Value obtained by means of Fisher’s Exact Test (p < 0.05).

## Discussion

The aim of the present study was to investigate the incidence of fracture of ProDesign Logic system instruments (Easy Equipamentos Odontológicos), in endodontic treatments performed by a specialist, in addition to identifying the dental group, arch, and root canal thirds in which they most frequently occurred. Considering the total number of teeth (n. 561) and canals treated (n. 1302), the overall fracture rates were 8.5 and 3.69%, respectively. As the number of canals varied according to the tooth, to establish the fracture incidence, the total number of canals must be considered^26,27^.

The ProDesign Logic system instruments (Easy Equipamentos Odontológicos) are made by means of machining the conventional NiTi alloy that is later submitted to a thermal treatment (CM—*controlled memory*). The aim of this process is: (i) to promote greater flexibility and resistance to cyclic and torsional fatigue of the files^23^; (ii) to conduct more conservative preparations, based on preservation of a larger quantity of peri-cervical dentin, and; (iii) to maintain the original root canal anatomy^24,25^. The instruments present accentuated distortion of the helices of the conical helical cutting stem, which reduce adherence of their cutting blades to the dentinal walls, thereby minimizing the chances of immobilization and torsional fracture^19,28^. However, the overall fracture rate observed in the present study (3.69%) was considerably higher than those identified in previous researches, in which treatments were also performed by specialists, but with the use of instruments made of conventional NiTi alloy—Protaper Universal (Dentsply-Maillefer)—1.10%^26^ and Profile 0.04 (Dentsply-Maillefer)—1.44%^29^. In the study by Al-Fouzan^29^, Profile 0.04 system kits (Dentsply-Maillefer) with instruments from #20 to #40 were used in up to 5 molars. Wu et al.^26^, used instruments from the Protaper Universal system (Dentsply-Maillefer) to perform preparations in a maximum of 3 molars, 10 premolars or 30 anterior teeth. In the present study, each instrument was used in up to 4 molars, 10 premolars with 2 canals, 20 premolars or 25 anterior teeth with a single canal. Furthermore, in the study by Wu et al.^26^, for example, instruments used in atresic and/or severely curved canals were used only once. In the present study and in the two above-mentioned studies^26,29^, all instruments were inspected with a magnifying loupe before, during and after use, and in case of deformation, they were immediately discarded. Despite being made of the same NiTi alloy, the use of different systems per se*,* represented an important aspect for the occurrence of fracture rates that differed substantially. Furthermore, the fracture of NiTi instruments in clinical practice is a “multifactorial phenomenon” influenced by a series of factors such as number of uses, design, speed, torque and root canal anatomy^16–18^.

In regard to the dental group, the majority of fractures occurred in molars (91.70%). Similar results were found by Wolcott et al.^30^ (94.70%), Wu et al.^26^ (94.30%) and Machado et al.^31^ (93.30%). The greater anatomical complexity inherent to this dental group led to predisposal to a greater possibility of this accident occurring^31–35^. Among the fractures observed in this study, 35.40% occurred in mandibular first molars. However, considering that these were the teeth most frequently treated (n. 89) this theoretically favored the higher rate of occurrence of the “event” studied. However, a similar number of maxillary first molars were also treated (n. 84) and the fracture rate was significantly lower (16.70%). In the mesial root of mandibular molars, in addition to the distal curvature that is often identified, buccolingual curvatures which are not seen in radiographs may also occur^36,37^. The occurrence of double curvatures made a significant contribution to the fracture of NiTi endodontic files by rotary flexion^16–18^.

When comparing the arches, there was no statistical difference in instrument fractures in the middle and apical root canal thirds (p = 0.307). However, this occurred more frequently in the apical third of both arches (p = 0.000). In the final millimeters of the root canal, NiTi instruments were more prone to fracture, either by torsion or by rotational flexion, due to their smaller dimensions and the presence of curvatures^16–18^.

As this was the first study conducted to investigate the incidence of fractures of ProDesign Logic system instruments (Easy Equipamentos Odontológicos) in primary endodontic treatments, this made it unfeasible to conduct specific comparative critical analyses. However, the high fracture rate observed in this study, when compared with values found in similar papers, should not be attributed only to the “intrinsic factors” previously discussed. The educational context must also be taken into consideration. In Brazil, specialization courses usually take place once a month for 3 or 4 days and last between 18 and 24 months. In contrast, residency programs in Endodontics at American universities, for example, are full-time and last for about 3 years. Therefore, students are subjected to a substantially higher training load, which provides them with more knowledge and clinical experience^31^. However, Ehrhardt et al.^38^, evaluated the quantity of fractures of Mtwo system instruments (VDW, Munich, Germany) in 556 endodontic treatments performed on molars and premolars by 6 Brazilian specialists, and identified an overall fracture rate of 1.98% (considering the number of teeth evaluated). This rate would be even lower if the number of canals treated were considered, but the authors did not disclose this information. Considering this result and the limitations of retrospective cross-sectional studies such as this investigation, it would appear that further researches are needed (especially prospective randomized clinical studies)^27^ to assess the fracture rates of ProDesign Logic system instruments (Easy Equipamentos Odontológicos) in a more reliable way.

## Conclusions

Despite the limitations of the present study, it could be concluded that the incidence of fracture of ProDesign Logic system instruments (Easy Equipamentos Odontológicos) was relatively high and more frequently observed in the apical third of molars.
